# CHA_2_ DS_2_ -VASc score, a simple clinical tool for early prediction of no-reflow phenomenon in patients undergoing emergency percutaneous coronary revascularization

**DOI:** 10.34172/jcvtr.2022.19

**Published:** 2022-06-25

**Authors:** Abdul Hakeem Shaikh, Rajesh Kumar, Ali Ammar, Afzal Hussain, Muhammad Naeem Mengal, Kamran Ahmed Khan, Danish Qayyum, Jawaid Akbar Sial, Tahir Saghir, Musa Karim

**Affiliations:** National Institute of Cardiovascular Diseases (NICVD), Karachi, Pakistan

**Keywords:** Primary Percutaneous Coronary Intervention, ST-segment Elevation Myocardial Infarction, Slow/No-Reflow, CHA2DS2-VASc

## Abstract

***Introduction:*** Slow flow/no reflow (SF/NR) phenomenon during emergency percutaneous revascularization is a feared complication associated with increased risk of adverse outcomes. CHA_2_ DS_2_ -VASc score has been proposed for the risk stratification but a very limited evidences are available regarding the accuracy of this system. Therefore, we conducted this study to assess the predictive value of CHA_2_ DS_2_ -VASc score for predicting SF/NR phenomenon during primary percutaneous coronary intervention (PCI).

***Methods:*** This analytical cross-sectional study included 596 consecutive patients undergoing PCI for STEMI at a tertiary care cardiac center of Karachi, Pakistan. Baseline -VASc sore was calculated and development of SF/NR phenomenon during primary PCI was recorded. Predictive value of the score was assessed through area under the curve (AUC) of receiver operating characteristic curve analysis and sensitivity and specificity were computed. Logistic regression analysis was performed to assess the predictive strength of the score.

***Results:*** A total of 596 patients were included, mean age was 56.28±11.44 years, and 75.7%(451) were male. The slow/no reflow phenomenon during the procedure was observed in 36.6%(218) of the patients. CHA_2_ DS_2_ -VASc≥2 was observed in 50.2%(299) of the patients. The CHA_2_ DS_2_ -VASc score was significantly higher in SF/NR patients, 2.06±1.25 vs. 1.37±1.33; *P*<0.001. The AUC of CHA_2_ DS_2_ -VASc score was 0.652 [0.607-0.696], CHA_2_ DS_2_ -VASc≥2 had sensitivity and specificity of 65.6% [58.9% to 71.9%] and 58.3% [53.6% to 63.7%] respectively for predicting SF/NR. CHA_2_ DS_2_ -VASc≥2 was insignificant on multivariate with odds ratio of 1.48 [0.72 -3.04]; *P*=0.283.

***Conclusion:*** CHA_2_ DS_2_ -VASc risk stratification system has moderate discriminating power for the stratification of SF/NR phenomenon during primary PCI.

## Introduction

 The emergency percutaneous revascularization within 12 hours of onset of symptoms remains the treatment of choice for patients with ST-segment elevation myocardial infarction (STEMI).^1,2^ A significant reduction in short- and long-term mortality of STEMI patients has been witnessed by virtue of technical as well as materialistic advancements in stent technology and pharmacological therapies.^3,4^ However, microvascular obstruction in distal coronary artery resulting in poor myocardial perfusion, known as slow/no reflow (SF/NR) phenomenon, remains the common complication after primary percutaneous coronary intervention (PCI) with incidence rate ranging from 4 to 44%.^5-8^ It is found to be associated with increased risk of short- and long-term adverse events. Pathogenesis of the phenomenon is not clear but various mechanisms have been postulated such as microvascular spasm, distal micro-embolization of thrombus fragments, and endothelial swelling due to reperfusion and ischemic injury.^9-11^

 Various studies have reported multiple patient related as well as system related factors as the potential predictors of SF/NR phenomenon,^8,11-14^ but unfortunately accurate and reliable risk stratification of patients for SF/NR phenomenon remains a clinical challenge. Recently, utility of CHA_2_DS_2_-VASc score for the risk stratification of SF/NR phenomenon has been argued by some of the researchers.^6,7^ The CHA_2_DS_2_-VASc score has been a well-established scoring system for the assessment of thromboembolism in patients with atrial fibrillation,^15^ however, its individual components are also found to be associated with development of SF/NR phenomenon during revascularization. Hence, the CHA_2_DS_2_-VASc scoring system has reported to have good predictive value for prediction of SF/NR phenomenon, however, supported evidences are very limited and accuracy of the score greatly varies from one study to other with area under the curve (AUC) of 0.56 ^6^ in one study and 0.83 ^7^ in other. Therefore, this study was designed to assess the predictive value of CHA_2_DS_2_-VASc sore for predicting SF/NR phenomenon during primary PCI for patients with STEMI.

## Materials and Methods

 With the approval of the ethical review committee of institution, consecutive patients diagnosed with STEMI and undergone primary PCI within recommended 12 hours window period from onset of the symptom were included in this study. Prior to inclusion, consent regarding participation in the study was obtained from all the patients. Patients with graft vessel PCI and coronary artery dissection during procedure were excluded from the study. This analytical cross-sectional study was conducted at the National Institute of cardiovascular Diseases (NICVD), Karachi, Pakistan during study period of six months from 1^st^ August 2020 to 31^st^ January 2021.

 STEMI was diagnosed based on baseline electrocardiography (ECG) findings and detailed history of patients was taken regarding symptomology, presentation, and pre-disposing risk factors. All the patients managed as per the institutional protocols and guidelines and all the primary PCI procedures were performed by the experienced interventional cardiologists. The pre- and post-procedure management, in-hospital and at discharge, were same for of the all the patients, i.e. loading does of P2Y12 receptor inhibitor, aspirin, and weight adjusted unfractionated heparin as per the current guidelines to every enrolled patients for primary PCI.

 The CHA_2_DS_2_-VASc risk score was calculated based on scoring schema proposed by Lip et al^15^, at the time of presentation for all the patients. The seven parameter structure of CHA_2_DS_2_-VASc score was as following; a score of one was assigned to each of the criterion of if patient had history of heart failure(C), case of hypertension (H), case of diabetes mellitus (D), diagnosed with vascular disease (V), female gender, and age between 65 to 74 years and a score of two was assigned to each of history of stroke (S2) and age ≥ 75 years. The presence of SF/NR phenomenon was defined positive when there was an episode of thrombolysis in myocardial infarction (TIMI) flow rate two (II) or lower in the infarct-related artery, despite mechanical opening of culprit lesion, without any evidence of coronary artery dissection.

 Sample size for the study was calculated using method of sample size calculation based on the receiver operating characteristic (ROC) curve analysis taking area under the curve (AUC) statistics using the method of calculation defined by Hajian-Tilaki K et al^16^ With the an expected AUC of 0.72,^6^ 4% margin of error and 95% confidence level the required sample size was computed to be 340. In order minimize the observation bias the computed sample size by inflated by 75%.

###  Statistical analysis

 All the statistical analysis was performed using IBM SPSS version 21. Analysis methods included descriptive summary of collected data with the use of frequency and percentage for categorical response variables and mean ± standard deviation (SD) for continuous response variables summarized at overall level as well as for the two groups of patients categorized based on SF/NR phenomenon. The distribution of clinical and demographic characteristics were compared between the group of patients with and without SF/NR with the use of appropriate statistical methods such as Chi-square test or Fisher exact test when comparison is of categorical nature and independent sample t test or Mann-Whitney U test when comparison is of continuous nature. The predictive value of CHA_2_DS_2_-VASc score was assessed by performing the receiver operating characteristic (ROC) curve analysis taking SF/NR as a state variable and the AUC [95% confidence interval (CI)] was computed. The CHA_2_DS_2_-VASc score value with the maximum Youden Index (J statistic) was identified as an optimal threshold value of CHA_2_DS_2_-VASc for the risk stratification of SF/NR. Performance of the identified threshold value of CHA_2_DS_2_-VASc score for risk stratification of SF/NR was evaluated in terms of accuracy, sensitivity, positive predictive value, specificity, and negative predictive. Binary logistic regression, univariate and multivariable, analyses were performed with a dichotomous variable of SF/NR as dependent variable and available patient and procedure related factors as independent variables. Odds ratios (OR) along with 95% CI were reported for univariate and multivariable analysis. Statistical significance was taken as p value ≤ 0.05.

## Results

 A total of 596 patients with STEMI undergoing PCI were included, 75.7% (451) patients were male and mean ± standard deviation (SD) age was 56.28 ± 11.44 years. The mean total ischemic time was 384.8 ± 185.9 minutes, around 13.9% (83) of the patients were in Killip class III-IV at presentation, and 6.7% (40) patients were in cardiac arrest. The slow/no reflow phenomenon during the procedure was observed in 36.6% (218) patients. Comparison of characteristics of the patients with and without slow/ no-reflow during procedure are presented in Table 1. Group of patients who developed slow/ no-reflow during procedure were tend to have higher proportion of female, older in age, higher proportion of older (≥65 years) patients, intubation rate (28.4% vs. 13.0%), cardiac arrest (12.8% vs. 3.2%), hypertension, diabetes, and past history of PCI (12.8% vs. 5.8%).

**Table 1:** Characteristics of the patients stratified by slow flow/ no-reflow during procedure

**Characteristics**	**Total**	**Slow flow/ No-reflow during procedure**		***P*** **-value**
		**No**	**Yes**	
**Total (N)**	596	378	218	**-**
**Female**	24.3% (145)	21.4% (81)	29.4% (64)	0.03*
**Age (years)**	56.28 ± 11.44	54.73 ± 11.6	58.97 ± 10.65	<0.001*
<65 years	73.3% (437)	77.8% (294)	65.6% (143)	0.005*
65 to 75 years	22.7% (135)	18.8% (71)	29.4% (64)	
>75 years	4% (24)	3.4% (13)	5% (11)	
**Height (cm)**	168.5 ± 5.8	169.4 ± 5.7	167 ± 5.7	<0.001*
**Weight (kg)**	73.8 ± 8	74 ± 7.6	73.4 ± 8.8	0.340
**Total ischemic time (minutes)**	384.8 ± 185.9	362.5 ± 181.1	423.5 ± 188.1	<0.001*
**Chest pain to ER time (minutes)**	285.9 ± 171.1	270.9 ± 164.5	311.8 ± 179.4	0.005*
**Systolic blood pressure (mmHg)**	131.4 ± 27.2	133.3 ± 25.5	128.1 ± 29.6	0.022*
**Heart rate (bpm)**	86.8 ± 21.4	85.5 ± 20.4	89 ± 23	0.057
**Killip Class III or IV**	13.9% (83)	9.5% (36)	21.6% (47)	<0.001*
**Cardiac Arrest**	6.7% (40)	3.2% (12)	12.8% (28)	<0.001*
**Co-morbids**				
Previous PCI	8.4% (50)	5.8% (22)	12.8% (28)	0.003*
Hypertension	54.4% (324)	48.9% (185)	63.8% (139)	<0.001*
Smoking	30.9% (184)	36% (136)	22% (48)	<0.001*
Diabetes mellitus	39.1% (233)	32.5% (123)	50.5% (110)	<0.001*
Family history of IHD	2.5% (15)	2.6% (10)	2.3% (5)	0.792
CVA/TIA	1.8% (11)	1.1% (4)	3.2% (7)	0.06
Chronic kidney disease	1% (6)	1.1% (4)	0.9% (2)	0.868
Congestive heart failure	1.7% (10)	0.8% (3)	3.2% (7)	0.027*
Peripheral vascular disease	0.2% (1)	0% (0)	0.5% (1)	0.188
**LVEDP (mmHg)**	20 ± 6.7	18.4 ± 6.2	22.8 ± 6.6	<0.001*
**LVEF (%)**	38.3 ± 9.1	39.8 ± 8.8	35.7 ± 9.1	<0.001*
**Multi-vessel disease**	65.6% (391)	62.7% (237)	70.6% (154)	0.049*
**Pre-procedure TIMI flow<III**	88.3% (526)	81.5% (308)	100% (218)	<0.001*
**Thrombus Grade ≥ 4**	72.8% (434)	63.5% (240)	89% (194)	<0.001*
**Post-procedure TIMI III flow**	80% (477)	100% (378)	45.4% (99)	<0.001*
**CHA**_2_ **DS**_2_ **-VASc score**	1.62 ± 1.34	1.37 ± 1.33	2.06 ± 1.25	<0.001*
<2	49.8% (297)	58.7% (222)	34.4% (75)	<0.001*
≥2	50.2% (299)	41.3% (156)	65.6% (143)	
**In-hospital mortality**	5.2% (31)	1.9% (7)	11% (24)	<0.001*

Abbreviations: PCI, percutaneous coronary intervention; MI,myocardial infarction; IHD,ischemic heart diseases; CVA,cerebrovascular accident; TIA,transient ischemic attack; LVEF, left ventricular ejection fraction; LVEDP, left ventricular end-diastolic pressure; TIMI, thrombolysis in myocardial infarction

**Figure 1:**
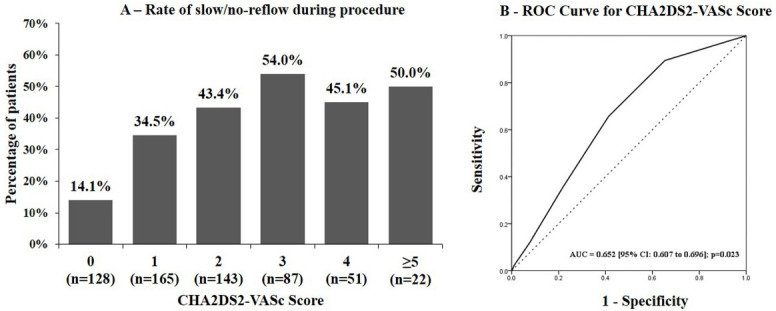
A; Rate of slow/no-reflow during procedure at various values of CHA_2_DS_2_-VASc score and B; ROC curve analysis for prediction of slow/no-reflow during procedure and

 Patients with slow/no-reflow during procedure were also tends to have higher proportion of TIMI flow grade < III at baseline (100% vs. 81.5%) and thrombus grade ≥ 4 (89.0% vs. 63.5%). The mean CHA_2_DS_2_-VASc score was 1.62 ± 1.34 with 50.2% (299) of the patients having score of ≥2. The mean CHA_2_DS_2_-VASc score was significantly higher for the patient with slow/ no-reflow during procedure, 2.06 ± 1.25 vs. 1.37 ± 1.33; p<0.001. The AUC of CHA_2_DS_2_-VASc score for predicting slow/ no-reflow was 0.652 [0.607 to 0.696] (Figure 1) and the optimal cutoff value was ≥2 with sensitivity of 65.6% [58.9% to 71.9%] and specificity of 58.3% [53.6% to 63.7%]. Accuracy of CHA_2_DS_2_-VASc score for prediction of slow/no-reflow during procedure is presented in Table 2.

**Table 2:** Accuracy of CHA_2_DS_2_-VASc score for prediction of slow flow/ no-reflow during procedure

**Characteristics**	**Total**	**CHA**_2_ **DS**_2_ **-VASc Score**		***P*** **-value**
		**< 2**	**≥ 2**	
**N**	**596**	**297**	**299**	**-**
**Slow flow/ No-relow during procedure**				
No	63.4% (378)	74.7% (222)	52.2% (156)	<0.001*
Yes	36.6% (218)	25.3% (75)	47.8% (143)	
**Diagnostic accuracy for assessment for slow flow/ no-relow during procedure**				
Accuracy		61.2% [95% CI; 57.2% to 65.2%]		
Sensitivity		65.6% [95% CI; 58.9% to 71.9%]		
Specificity		58.3% [95% CI; 53.6% to 63.7%]		
Positive Predictive Value		47.8% [95% CI; 44.0% to 51.7%]		
Negative Predictive Value		74.8% [95% CI; 70.6% to 78.4%]		

Abbreviation: CI, confidence interval *significant at 5%

 Univariate and multivariate logistic regression analysis to determine the predictors of slow/no-reflow phenomenon during procedure are presented in Table 3. Among the various characteristics of the patients the independent predictors of slow/no-reflow phenomenon based on multivariable analysis are total ischemic time of more than 7 hours, cardiac arrest, history of CVA/TIA, baseline LVEDP >20 mmHg, and thrombus grade of ≥ 4. CHA_2_DS_2_-VASc score of ≥ 2 was significant with OR of 2.71 [1.92 -3.84]; p<0.001 in univariate analysis but it was observed to be insignificant on multivariable analysis.

**Table 3:** Predictors of slow flow/ no-reflow during procedure (univariate and multivariable logistic regression)

**Factors**	**Univariate**		**Multivariable**	
	**OR [95% CI]**	***P*** **-value**	**OR [95% CI]**	***P*** **-value**
Female	1.52 [1.04 -2.23]	0.03*	1.08 [0.66 -1.77]	0.766
≥ 65 years	1.84 [1.27 -2.66]	0.001*	1.43 [0.89 -2.31]	0.143
TIT >7 hours	1.87 [1.32 -2.63]	<0.001*	1.52 [1.02 -2.26]	0.037*
Killip class (III or IV)	2.61 [1.63 -4.18]	<0.001*	0.99 [0.55 -1.76]	0.966
Cardiac Arrest	4.49 [2.24 -9.04]	<0.001*	2.9 [1.31 -6.43]	0.009*
Previous PCI	2.38 [1.33 -4.28]	0.004*	1.2 [0.59 -2.43]	0.613
Hypertension	1.84 [1.3 -2.58]	<0.001*	1.19 [0.73 -1.95]	0.483
Smoking	0.5 [0.34 -0.74]	<0.001*	1.42 [0.85 -2.36]	0.176
Diabetes mellitus	2.11 [1.5 -2.97]	<0.001*	4.42 [1.04 -18.7]	0.044*
CVA/TIA	3.1 [0.9 -10.72]	0.074	0.42 [0.06 -3.15]	0.397
Chronic kidney diseases	0.87 [0.16 -4.77]	0.868	1.35 [0.3 -6.14]	0.696
Congestive heart failure	4.15 [1.06 -16.21]	0.041*	2.43 [1.56 -3.78]	<0.001*
LVEDP >20 mmHg	0.28 [0.19 -0.4]	<0.001*	1.25 [0.79 -1.98]	0.341
LVEF <35%	3.63 [2.51 -5.23]	<0.001*	4.47 [2.66 -7.52]	<0.001*
Thrombus Grade ≥ 4	4.65 [2.9 -7.46]	<0.001*	1.48 [0.72 -3.04]	0.283
CHA_2_DS_2_-VASc score ≥ 2	2.71 [1.92 -3.84]	<0.001*	1.08 [0.66 -1.77]	0.766

Abbreviations: PCI, percutaneous coronary intervention; TIT,total ischemic time; TIA,transient ischemic attack; CVA,cerebrovascular accident; LVEF, left ventricular ejection fraction; LVEDP, left ventricular end-diastolic pressure; CI,confidence interval; OR,odds ratio *significant at 5%

## Discussion

 This study was conducted with aim of to assess the predictive value of CHA_2_DS_2_-VASc score for prediction of slow/no-reflow phenomenon during primary PCI, we observed that CHA_2_DS_2_-VASc score has moderate accuracy in predicting slow/no-reflow phenomenon with AUC of 0.652 [0.607 to 0.696] and sensitivity and specificity of 65.6% [58.9% to 71.9%] and 58.3% [53.6% to 63.7%] respectively at cutoff value of ≥2. Odds ratio of CHA_2_DS_2_-VASc score ≥ 2 for slow/no-reflow was significant on univariate analysis but it was insignificant on multivariable analysis. The independent predictors of slow/no-reflow phenomenon during the procedures were observed to be the total ischemic time of higher than 7 hours, cardiac arrest, history of CVA/TIA, baseline LVEDP of >20 mmHg, and thrombus grade of ≥ 4.

 Contrary to our observation, a study conducted by Mirbolouk F et al^7^ reported good predictive power of CHA_2_DS_2_-VASc score with AUC of 0.83 [0.79 to 0.88] and sensitivity and specificity of CHA_2_DS_2_-VASc score at cutoff value of ≥2 was reported to be 88% and 67% respectively. Furthermore study reported potential of CHA_2_DS_2_-VASc score as independent predictor alongside other independent predictors such as higher diastolic blood pressure, lower systolic blood pressure, smaller stent size, and pre- TIMI flow grade 0. However, this was a retrospective study with a small sample size. Another retrospective study by Ashoori A et al^6^ however reported AUC of 0.56 [0.495 to 0.631] for of CHA_2_DS_2_-VASc for discrimination of no-reflow.

 Our findings were more align with the prospective study by Ipek G et al^17^ which included 2375 consecutively selected patients and observed AUC of 0.63 [0.57 to 0.70] for the prediction of no-reflow with sensitivity and specificity of 66% and 59% respectively at cutoff value of CHA_2_DS_2_-VASc score ≥2. Other independent predictors of no-reflow reported in this study were stent length, lower stent diameter, anterior MI, and KILLIP classification. Lacking of potentially more significant predictors of no-reflow in the calculation of CHA_2_DS_2_-VASc score, such as ischemic time, thrombus grade, LVEDP, and KILLIP classification, can be a primary mechanism behind insignificance of CHA_2_DS_2_-VASc score in multivariable analysis of no-reflow.

 Association of SF/NR with adverse clinical course of patients is already well documented in multiple past studies,^8,12,14,18^ our observations regarding prognostic role of SF/NR were similar to what already reported in past studies, in-hospital mortality rate was observed to be significantly higher with mortality rate of 11% vs. 1.9%; p<0.001 for the patients with and without SF/NR during primary PCI respectively. Although the mechanism of development of SF/NR is not clear, but multiple factors have been reported to be associated with it, the most commonly observed characteristics were advanced age (>65 years),^8,12,19,20^ longer lesions,^8,12,19-21^ low pre-procedure TIMI flow grade,^8,12,18-21^ longer ischemic time,^12,18-20^ and thrombus grade.^12,14,18-20^

 Even though CHA_2_DS_2_-VASc score has been reported as an independent predictor of slow/no-reflow phenomenon^6,7,17^ but in this study on multivariate analysis CHA_2_DS_2_-VASc score failed to meet the set significance criteria. Although, it is easily applicable scoring system, but it has moderate discriminating power for categorization of patients at high risk of development of slow/no-reflow during procedure, therefore, it is imperative to develop a more accurate and dedicated risk stratification system of slow/no-reflow phenomenon. Additionally, the prolonged ischemic time was found to be an independent predictor of SF/NR phenomenon in our population, here we observed a significant out of hospital delay with an average of 285.9 ± 171.1 minutes. Past studies in our population have reported unawareness of symptomology and unavailability of transportation as the two major factors for pre-hospital delay.^22^ Mass awareness programs regarding typical cardiac symptoms as well as initiatives to create ease of access to the healthcare system need to be ensured by the concern governmental and non-governmental authorities.

 A single center experience with relatively small sample size are the key limitations of this study. Additionally, slow/no-reflow was estimated only on available angiographic findings which were not long enough to be measured by more authentic tool like myocardial blush grading (MBG).

## Conclusion

 In conclusion, CHA_2_DS_2_-VASc score is an easy to use risk stratification system with moderate discriminating power for the stratification of patients at high risk of development of slow/no-reflow phenomenon during primary PCI. More research efforts are needed in this area to formulate a dedicated more accurate risk stratification scoring system for slow/no-reflow phenomenon based on patient and system related predictors such as co-morbid conditions, age, ischemic time, pathophysiologically characteristics, and lesion characteristics.

## Acknowledgements

 The authors wish to acknowledge the support of the staff members of the Clinical Research Department of the National Institute of Cardiovascular Diseases (NICVD) Karachi, Pakistan.

## Funding

 None.

## Competing interests

 The authors declare that they have no conflicts of interest.

## Ethical approval

 This study was approval by the ethical review committee of the National Institute of Cardiovascular Diseases (NICVD), Karachi (ERC-35/2020).
